# Thalamostriatal degeneration contributes to dystonia and cholinergic interneuron dysfunction in a mouse model of Huntington’s disease

**DOI:** 10.1186/s40478-020-0878-0

**Published:** 2020-02-07

**Authors:** Gabriel Crevier-Sorbo, Vladimir V. Rymar, Raphael Crevier-Sorbo, Abbas F. Sadikot

**Affiliations:** Department of Neurology and Neurosurgery, Montreal Neurological Institute, McGill University, 3801 University street, H3A 2B4, Montreal, Quebec Canada

**Keywords:** Parafascicular, Striatum, Thalamus, R6/2, Basal ganglia, Immunotoxin

## Abstract

Huntington’s disease (HD) is an autosomal dominant trinucleotide repeat disorder characterized by choreiform movements, dystonia and striatal neuronal loss. Amongst multiple cellular processes, abnormal neurotransmitter signalling and decreased trophic support from glutamatergic cortical afferents are major mechanisms underlying striatal degeneration. Recent work suggests that the thalamostriatal (TS) system, another major source of glutamatergic input, is abnormal in HD although its phenotypical significance is unknown. We hypothesized that TS dysfunction plays an important role in generating motor symptoms and contributes to degeneration of striatal neuronal subtypes. Our results using the R6/2 mouse model of HD indicate that neurons of the parafascicular nucleus (PF), the main source of TS afferents, degenerate at an early stage. PF lesions performed prior to motor dysfunction or striatal degeneration result in an accelerated dystonic phenotype and are associated with premature loss of cholinergic interneurons. The progressive loss of striatal medium spiny neurons and parvalbumin-positive interneurons observed in R6/2 mice is unaltered by PF lesions. Early striatal cholinergic ablation using a mitochondrial immunotoxin provides evidence for increased cholinergic vulnerability to cellular energy failure in R6/2 mice, and worsens the dystonic phenotype. The TS system therefore contributes to trophic support of striatal interneuron subtypes in the presence of neurodegenerative stress, and TS deafferentation may be a novel cell non-autonomous mechanism contributing to the pathogenesis of HD. Furthermore, behavioural experiments demonstrate that the TS system and striatal cholinergic interneurons are key motor-network structures involved in the pathogenesis of dystonia. This work suggests that treatments aimed at rescuing the TS system may preserve important elements of striatal structure and function and provide symptomatic relief in HD.

## Introduction

Huntington’s disease (HD) is a progressive autosomal dominant neurodegenerative disorder characterized by choreiform movements, dystonia and psychiatric symptoms [1, 2]. HD is caused by an abnormal expansion of CAG trinucleotides in exon 1 of the *huntingtin* gene (*mhtt*) with larger numbers of repeats leading to earlier age of onset and more severe symptoms [3]. Despite ubiquitous expression of *mhtt*, medium spiny projection neurons (MSNs) of the striatum are a major target for degeneration [2]. Interneurons, which comprise a small proportion of all striatal neurons, were initially thought to be spared in HD [4]. However recent work suggests that the density of GABAergic parvalbumin (PV) positive [5] and cholinergic interneuron subtypes are reduced in HD [6] with relative sparing of other interneuron groups [5–7]. Multiple pathophysiological mechanisms may explain the predilection for striatal neuronal loss including: hyperexcitability, loss of afferent-derived trophic support, immune cell activation, and diverse intracellular signaling abnormalities [8–16].

Loss of afferent-mediated trophic support contributes to neuronal loss in common neurodegenerative diseases, including Alzheimer’s disease [17, 18] and Parkinson’s disease [19–21]. Trophic support from the major glutamatergic striatal afferent systems may also play an important role in HD. In vivo imaging and autopsy studies suggest that the cerebral cortex atrophies in prodromal HD along with the striatum, and this atrophy is severe by late stages [2, 22]. Recent MRI studies indicate that the thalamus also undergoes significant atrophy in early disease [23]. *Post-mortem* analysis indicates that the posterior intralaminar thalamus, or centromedian-parafascicular (CM-PF) complex, is an important target for degeneration in HD [24].

The CM-PF in primates or the parafascicular (PF) in rodents is a major source of glutamatergic afferents to the striatum, specifically targeting MSNs of the matrix sub-compartment of the neostriatal mosaic [25–27]. The PF also provides dense input to two major striatal interneuron subtypes implicated in HD: the cholinergic and PV positive interneurons [28–30]. Recent ultrastructural studies in the heterozygous Q140 mouse model of HD suggest early pathology in the thalamostriatal (TS) projection prior to corticostriatal degeneration [31, 32]. Further experiments show coexistent ultrastructural pathology of striatal cholinergic interneurons at early time-points in Q140 mice [31].

In order to determine whether thalamic inputs to the striatum play a critical role in survival of striatal neurons and in development of motor dysfunction in HD, we assessed the structural and functional effects of early PF lesions in an animal model of HD. We used the R6/2 model, a transgenic mouse with approximately 125 CAG repeats in the N-terminal portion of the *mhtt* gene [33]. The R6/2 mouse is a well-studied model and reproduces many of the motor and morphological features of HD [34]. Our results provide evidence for early degeneration of PF neurons prior to striatal neuron loss in the R6/2 model. Early lesions of the TS in R6/2 mice result in an acceleration of clasping movements suggesting worsened dystonic behaviour. PF lesions do not accelerate the time course of progressive loss of spontaneous locomotion in an open field during the R6/2 lifespan. PF lesioned mice regardless of genotype show decreased exploration using the contralateral forelimb. Morphological analysis indicates that PF lesions do not alter the extent of degeneration of striatal projection neurons and PV neurons in R6/2 mice. In contrast, TS lesions in R6/2 mice lead to early degeneration of striatal cholinergic neurons. Finally, early unilateral striatal cholinergic ablation in R6/2 mice using cell-specific immunotoxins also leads to an increase in clasping suggesting an important link between TS inputs to cholinergic neurons and dystonia in HD.

## Materials and methods

### Animals

The behavioural experiments were performed using R6/2 mice and WT littermate mice from a colony maintained at the Facility for Neurological Disease Models of the Montreal Neurological Institute. Ovarian transplanted R6/2 females were obtained from a line maintained at The Jackson Laboratory and were crossed with males of the C57BL6J background. CAG repeat lengths were sequenced and found to be between 119 and 125 for R6/2 mice and normal for WT littermates.

### Surgery and lesion verification

All surgical procedures were performed in accordance with the Standard Operating Procedures (SOPs) for stereotaxic mouse surgery at McGill University. Twenty-eight day-old mice were anaesthetized using a ketamine and xylazine (Rompun, Bayer, USA) cocktail**.** Stereotactic lesions were made at coordinates corresponding to the PF (Bregma − 2.20 mm, − 3.3 mm below the cortical surface, and 0.6 mm lateral to midline) [35]. A loop-shaped retractable leucotome [36] was inserted to the level of the PF, deployed to a radius of 0.5 mm, rotated twice, closed and then retracted. Sham-lesioned animals underwent the same procedure except the leucotome was inserted 2.5 mm beneath the cortical surface but not deployed. Lesions were verified on Nissl stain or Nissl-NeuN using the 4X objective and the extent of each lesion was analyzed on images captured on tiled images (StereoInvestigator (v10, Microbrightfield, USA). Mice with lesions that either crossed the midline or with large lesions extending beyond the PF into the ventral thalamus were excluded.

### Saporin injection and verification of effects in striatum

Use of anti-ChAT conjugated saporin toxins are well-described for selectively ablating cholinergic interneurons in the rodent striatum [37]. Using the same stereotactic techniques mentioned above, 28-day-old R6/2 and WT mice underwent unilateral, striatal injections with either anti-ChAT-saporin or Rabbit IgG-saporin (ATS BIO, USA). The total volume and concentration of either saporin construct was the same (0.7 μL of 0.6 μg/μL solution). The approximate center of mass of the neostriatum was targeted (0.65 mm from Bregma, 2.6 mm from the cortical surface and 2.15 mm lateral to midline) [35]. The toxin was infused at a rate of 0.1 μL /minute using an automated system (Pump 11 Elite, Harvard Apparatus, USA) through a 5 μL syringe (Hamilton 700 series, USA). Histological sections were immunostained for ChAT protein and counterstained with cresyl violet allowing visualization of the needle tract, confirming injection placement in the neostriatum, and allowing unbiased stereological analysis of striatal cholinergic cell morphology.

### Behavioural studies

All behavioural testing was performed during the first five hours of the light phase in a standard 12-h light–dark cycle [38]. Tests were performed at 4, 6, 9 and 11 wks ± 1 day (Additional file 1, Experimental Timeline), with the open field and cylinder test at day 1, and the clasping test at day 2 [38].

### Spontaneous locomotion in open field

Mice were placed in a four arena 50X50cm open field with infrared backlighting for one-hour [34], and movements were videotaped using an overhead camera [39] and later analyzed using VideoTrack (Viewpoint, Montreal, Canada). Spontaneous voluntary locomotor activity was categorized as follows: inactivity or non-ambulatory movements (< 1 cm/second), moderate speed (between 1 and 5 cm/second) or fast speed (> 5 cm/second).

### Vertical exploratory behaviour

Mice were placed in a plexiglass cylinder (diameter 20 cm, height 30 cm) with two mirrors positioned behind the cylinder in order to ensure a 360-degree view of the animal’s forelimb wall touches. The session was video-recorded and the number of vertical contacts on the cylinder wall with the right paw, left paw or both paws simultaneously were scored on frame by frame analysis with the viewer blind to operative status and genotype.

### Clasping score

A tail suspension or clasping test was used to assess the development of dystonic forelimb contractions previously documented in the R6/2 mouse [14, 34]. Mice were suspended by the tail at a height of at least 30 cm, for three trials lasting 30-s each, while limb movements were videotaped. Clasping was defined as a retraction of a limb toward the body. In order to provide a semi-quantitative index of abnormal involuntary movements, clasping at each limb was graded as: none = 0, mild = 0.25, moderate = 0.5, severe = 0.75 by an observer blind to genotype. Clasping was rated as: “none” if the mouse did not retract the limb towards the midline and “mild” if partial retraction of a limb occurred toward the midline but did not reach the midline, and the contraction was not sustained. “Moderate” clasping was a high-amplitude limb retraction to or beyond the midline that was not sustained, or partial limb retraction that was sustained for > 5 consecutive seconds. “Severe” clasping was a high-amplitude limb retraction to or beyond the midline sustained for > 5 s. The score for the forelimbs and hindlimbs was summed making the maximum score 3. The average value of all three clasping trials was analyzed.

### Tissue processing

R6/2 mice and WT mice were deeply anesthetized and perfused transcardially with 0.9% heparinized saline followed by 4% paraformaldehyde in phosphate buffer (4% PFA) (0.1 M, pH 7.4) both at 4 °C. Brains were removed, fixed in 4% PFA for 24 h then transferred to a phosphate buffered 30% sucrose solution for 24–48 h. Brains were sectioned at 40 μm in the coronal plane with a freezing microtome. Free-floating sections were collected serially in six vials containing phosphate-buffered saline (PBS, 0.1 M, pH 7.4). One set of sections was mounted out of distilled water onto glass slides, stained in 0.1% cresyl violet (Nissl stain) and coverslipped using Permount (Fisher Scientific, Whitby, ON, Canada). The remaining vials were immediately placed in buffered anti-freeze solution and stored at − 20 °C.

### Immunohistochemistry

The following primary antibodies were used in these experiments: mouse anti- NeuN (Millipore, Etobicoke, Canada; MAB377, 1:1000), Rabbit anti-μ-opioid receptor (Immunostar, Hudson, USA; #24216; 1:8000), mouse anti-parvalbumin (Swant, Fribourg, Switzerland; #235; 1:5000) and rabbit anti-ChAT (Millipore; AB143; 1:600). Sections were removed from antifreeze, rinsed six times in PBS, and then incubated for one hour in a blocking solution (10% bovine serum albumin (BSA), 0.3% Triton-X, 0.1 M PBS, pH 7.4). Next the sections were incubated in primary antibody in PBS containing 0.1% Triton-X and either 2% BSA or 5% NGS for 24–48 h at 4 °C. After washes in PBS, sections were incubated in the following biotinylated secondary antibodies: horse anti-mouse IgG (Vector Laboratories, Burlingame, California, USA; BA-2000; 1:200), goat anti-rabbit IgG (Vector Laboratories; BA-1000; 1:200). Sections were washed once more in PBS and then incubated for 1 h in 1:100 ABC elite kit (PK6100, Vector Laboratories). Antibody binding was revealed using 0.05% 3,3′-diaminobenzidsine (D5905, Sigma-Aldrich, Oakville, ON, Canada) in TBS (pH 7.6) and hydrogen peroxide (0.01%). All slices were then mounted out of distilled water onto slides, counterstained with 0.1% cresyl violet and coverslipped using Permount (SP15, Fisher Scientific).

### Unbiased stereology

An unbiased stereological probe, the optical fractionator [40], was used to estimate the number of neurons in the areas of interest. The stereology apparatus consisted of a light microscope (BX40, Olympus, Japan) coupled with a video camera (DC200, DAGE, USA), motorized X–Y stage (BioPoint XYZ, LEP, USA), Z-axis indicator (MT12 microcator, Germany), and a computer running Stereo Investigator software (v11.06.2, Microbrightfield, USA). The neostriatum was delineated according to previously defined boundaries [34] using the mouse brain atlas of Paxinos and Franklin [35] and a 4X objective. Rostral and caudal limits were determined by the first and last coronal sections with visible caudate–putamen (Bregma 1.7 mm to − 2.0 mm) [35]. Every sixth serial histological section within this zone was examined (240 μm intervals). The dorsal, medial and lateral limits of the neostriatum are well defined in the mouse brain atlas [35]. The ventral limit of the striatum at the post-commissural part is well delineated on Nissl stains. At the pre-commissural levels, we delimit the dorsal striatum from the nucleus accumbens with a line that extends from above the ventral most part of the lateral ventricle medially, to the tapered external capsule laterally, at an angle of 25–30° below the axial plane [34, 41]. The PF was delineated using the same mouse brain atlas [35] using a 10X objective. All sections with a clearly distinguishable PF were delineated (Bregma − 2.0 mm to − 2.5 mm) [35]. Every other section within the PF reference range was examined (80 μm intervals).

Systematic random sampling of neurons was performed by randomly translating a grid onto the section of interest. At each intersection of grid lines an optical fractionator counting frame with exclusion lines was applied. A 150X150 μm grid size and a 60X60 μm counting frame was used for the PF neuron optical fractionator analysis (Gunderson CE (m = 1) = 0.038 ± 0.001). A 300X300 μm grid size and a 25X25 μm counting frame was used for optical fractionator analysis in the neostriatum (Gunderson CE (m = 1) = 0.029 ± 0.001). A 250X250 μm grid size and a 70X90 μm counting frame was used for the parvalbumin interneuron optical fractionator analysis (Gunderson CE (m = 1) = 0.064 ± 0.001). A 175X175 μm grid size and a 70X90 μm counting frame was used for the cholinergic interneuron optical fractionator analysis (Gunderson CE (m = 1) = 0.080 ± 0.003). All randomly assigned sample sites were then examined using a 100X objective (oil; numerical aperture, 1.3). Section thickness was assessed every ten counting sites using the Z-axis indicator (MT12 microcator, Germany). The top of the neuron was used as a unique identifier in all analyses. Neurons falling in the counting frame were counted only if they came into focus within a predetermined 8-μm-thick optical dissector positioned 1-μm above and below the surface of the mounted section as indicated by the Z-axis indicator. For the neostriatal mosaic analysis, neurons were distinguished on Nissl-stains based on cell diameter (> 7 μm), and a lighter cytoplasm containing a dense nucleus [34].

Neuron soma area and volume of the PF and striatum were estimated using a four-ray nucleator probe [42] or the Cavalieri probe [40] respectively. For the Cavalieri probe, a grid of 40X40 μm squares was randomly translated over the delineated structures of interest and markers were placed at the intersection of grid lines that fell within the delineated structure. Estimates of the total number of neurons, soma area and Cavalieri volume were calculated by the Stereo Investigator software (v10, Microbrightfield, USA).

### Statistical analyses

Normality was assessed prior to performing comparative tests using the Shapiro-Wilks test. An analysis of variance (ANOVA) was performed on normal data using the aov function in R [43]. Post hoc analysis of normal data consisted of a two-tailed, paired or unpaired t-test based on whether the samples were dependent or independent respectively. Post hoc tests on normal data were corrected for multiple comparisons using *Tukey’s Honestly Significant Difference* test (HSD). A non-parametric ANOVA was performed on non-normally distributed data or ordinal data using the art function from the ‘ARTool’ package [44] in R. Post hoc analysis for non-parametric data included a two-tailed Mann-Whitney U-test or a Wilcoxon signed-rank test for independent and dependent samples respectively. Non-parametric post hoc tests were corrected for multiple comparisons using the *Bonferroni* correction. For behavioural tests, the main factors of the ANOVA were time as a within-subject factor, and genotype and lesion status as between-subject factors. The main ANOVA factors for morphological studies, genotype and lesion status were analyzed as independent groups. All data are expressed as averages ± standard error of the mean (SEM). The SEM is represented graphically as error bars. *P*-values ≤0.05 were considered significant.

## Results

### The PF degenerates in the R6/2 model of HD

**Fig. 1:**
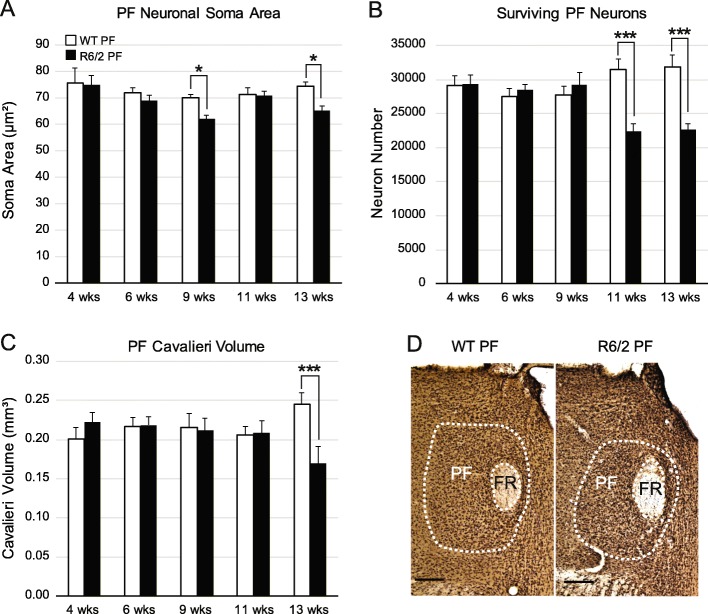
Morphology of the PF nucleus over the R6/2 mouse lifespan compared to WT controls. (**a**) There is a significant decrease in neuronal area at 9 (*p* = 0.03) and 13 wks (*p* = 0.01) in R6/2 compared to WT mice, based on analysis with the nucleator, an unbiased stereology probe. (**b**) Loss of PF neurons in R6/2 compared to WT mice at 11 wks (*p* = 0.0001) and 13 wks (*p* = 0.0002) determined using the optical fractionator, an unbiased stereology probe. (**c**) Reduction in PF volume is noted at 13 wks compared to WT mice determined using the Cavalieri probe (*p* = 0.0007). (**d**) Photomicrographs of NeuN/Nissl stained coronal sections outlining the PF nucleus in WT and R6/2 mice at 13 wks. Scale bar: 250 μm. The data sets were analyzed using a two-way between subject ANOVA and a Tukey HSD post hoc test: *p < 0.05, *** *p* < 0.001. For all panels of Fig. 1, 4 week: WT (*n* = 3), R6/2 (*n* = 4); 6 week: WT (n = 4), R6/2 (*n* = 5); 9 week: WT (*n* = 4), R6/2 (*n* = 4), 11 week: WT (*n* = 6), R6/2 (*n* = 4); 13 week: WT (*n* = 5), R6/2 (*n* = 4). Abbreviations: FR = Fasciculus Retroflexus, PF = Parafascicular Nucleus

To determine if the PF is susceptible to degeneration in HD, we quantified neuron numbers and soma size in the PF throughout the lifespan of the R6/2 mouse model using unbiased stereology. Application of the nucleator probe demonstrated that soma size of PF neurons was reduced in R6/2 mice compared to WT at 9 and 13 weeks (wks) (Fig. 1a, w (43) = 0.954, *p* = 0.086, F(GenotypeXTime)_4,33_ = 1.45, *p* = 0.24, F(Time)_4,33_ = 3.85, *p* = 0.01, F(Genotype)_1,33_ = 8.46, *p* = 0.006, post hoc comparison: 9 wks *p* = 0.03 and 13 wks *p* = 0.01). Analysis using the optical fractionator probe revealed a significant 29% decrease in the number of PF neurons in R6/2 mice at 11 wks compared to WT (Fig. 1b, w (43) = 0.972, *p* = 0.37, F(GenotypeXTime)_4,33_ = 7.65, *p* = 0.00018, post hoc*:* 11 wks *p* = 0.00014; 13 wks *p* = 0.00015). Neuronal degeneration progressed at later timepotins, and by 13 wks the Cavalieri volume estimate of the PF was 31% smaller in R6/2 mice compared to WT at 13 wks (Fig. 1c-e, w (43) = .948, *p* = 0.05, F(GenotypeXTime)_4,33_ = 3.34, *p* = 0.021; post hoc *p* = 0.0007). In summary, the main source of TS projections, the PF, shows an early reduction in neuronal size in R6/2 mice at 9 wks, followed by progressive neuronal loss at 11 and 13 wks of age.

### The effect of PF lesions on motor behavior in R6/2 and WT mice

**Fig. 2:**
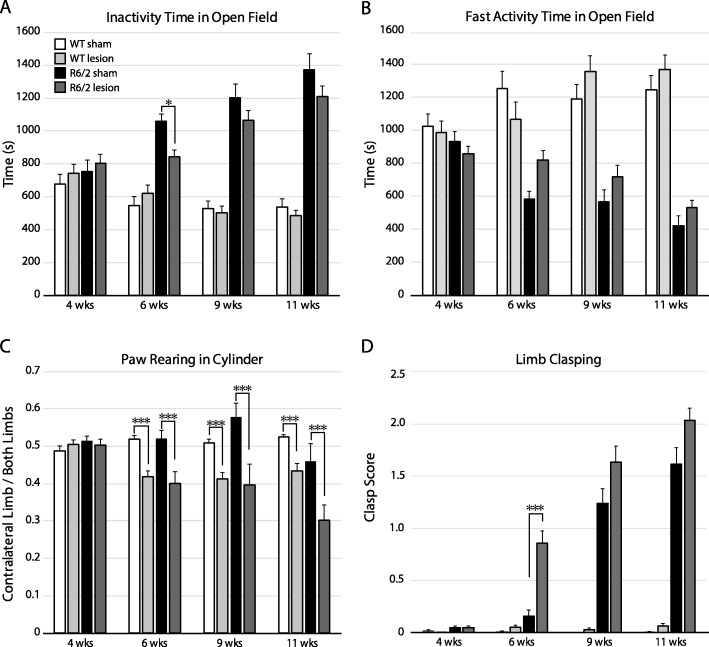
Characterization of motor behavior following unilateral PF lesions in R6/2 and WT mice. Time spent at rest (**a**) or moving rapidly (**b**) during a one-hour open field session demonstrating a transient decrease in rest time at 6 wks in R6/2 mice following PF lesions compared to sham-lesions (*p* = 0.01), that is not sustained at later time-points. (**c**) The cylinder test assessing voluntary paw reaching motor behaviour shows a persistent decrease in contralateral limb use in both WT and R6/2 mice after PF lesions (6 wks *p* = 0.00004, 9 wks *p* = 0.00004, 11 wks *p* = 0.00008). (**d**) A significant increase in dystonic clasping is noted in PF lesioned R6/2 compared to sham treated R6/2 mice at 6 wks (*p* = 0.00008). A 3-way non-parametric ANOVA was applied to each data set, followed by a Bonferroni post hoc correction; *p < 0.05, ***p < 0.001. For Fig. 2 a-d: WT sham: *n* = 18, WT lesion: *n* = 17, R6/2 sham: *n* = 15, R6/2 lesion: *n* = 22

The open field test assesses spontaneous voluntary locomotor activity [39]. To determine the effect of PF lesions on locomotor activity, R6/2 and WT mice were placed in an open field for one hour at 4, 6, 9 and 11 wks. In keeping with previous studies [34], we found a progressive increase in inactivity time over the R6/2 mouse lifespan starting at 6 wks in both sham-lesioned and lesioned groups compared to their respective WT groups (Fig. 2a, f (TimeXGenotypeXLesion)_3201_ = 2.82, *p* = 0.04, post hoc all *p* < 0.02 for sham R6/2 mice vs. WT sham as of the 6-week time-point). Lesioned R6/2 mice spent significantly less time resting at 6 wks compared to sham R6/2 mice (*p* = 0.01), but not at later time-points. Periods of fast movement time reflected inactivity time, with progressive decrease in locomotion in R6/2 compared to WT mice. There was a non-significant (*p* = 0.07) trend to increased locomotion at 6 wks in lesioned compared to sham-lesioned R6/2 mice (Fig. 2b, f (TimeXGenotypeXLesion)_3201_ = 4.64, *p* = 0.004). Thus, PF lesioned R6/2 mice progress to the same hypokinetic state with poverty of spontaneous voluntary movement as sham-lesioned counterparts.

The cylinder test assesses exploratory vertical paw reaching limb asymmetry, a complex voluntary behaviour requiring spatial sensorimotor coordination [45]. The number of paw touches on the walls of a cylinder were quantified during a five-minute session in PF lesioned and sham-lesioned WT and R6/2 mice at 4, 6, 9 and 11 wks. A significant reduction in the percent of contralateral limb touches occurs at all post-operative ages after PF lesions in both WT and R6/2 mice compared to sham counterparts (Fig. 2c, f (TimeXGenotypeXlesion)_3167_ = 0.43, *p* = 0.73, F(TimeXLesion)_3167_ = 13.4, *p* < 0.00001; F(TimeXGenotype)_3167_ = 4.82, *p* = 0.003, post hoc all *p* < 0.001). Thus, both R6/2 and WT mice preferentially explored vertical cylinder space with the ipsilateral limb following PF lesions.

The tail suspension test or clasping test is a widely used method for eliciting dystonic movements in HD and dystonia mouse models [34, 46, 47]. To determine if PF lesions affect the clasping phenotype, mice were tested prior to lesions and at three post-operative time-points. R6/2 mice had a worsening in dystonic clasping behaviour with aging in both sham and lesion groups with a significant increase in limb clasping in R6/2 mice at 6 wks following PF lesions compared to sham-lesioned R6/2 mice (Fig. 2d, f (TimeXGenotypeXLesion)_3210_ = 26.63, *p* < 0.00001, post hoc: 6-week R6/2 sham vs 6-week R6/2 lesion *p* = 0.00008). Virtually none of the WT mice exhibited clasping and PF lesions did not induce dystonic behaviour in this group. Thus, PF lesions significantly worsen the clasping phenotype in R6/2 mice.

### Striatal morphology after early PF lesions

Previous work in R6/2 mice using unbiased stereology on Nissl stained sections demonstrates that significant striatal cell loss and atrophy occurs at 11 and 13 wks [34]. To determine if the PF has a trophic role for striatal neurons faced with degenerative stress in HD, we quantified the number and soma size of striatal neurons at 11 and 13 wks following PF lesions at 4 wks of age. Since the posterior intralaminar nuclei preferentially afferent the matrix compartment of the striatal mosaic [25–27], neurons of the striosome and matrix compartments were analyzed separately using μ-opiate receptor (MOR) as a marker of striosomes.

**Fig. 3:**
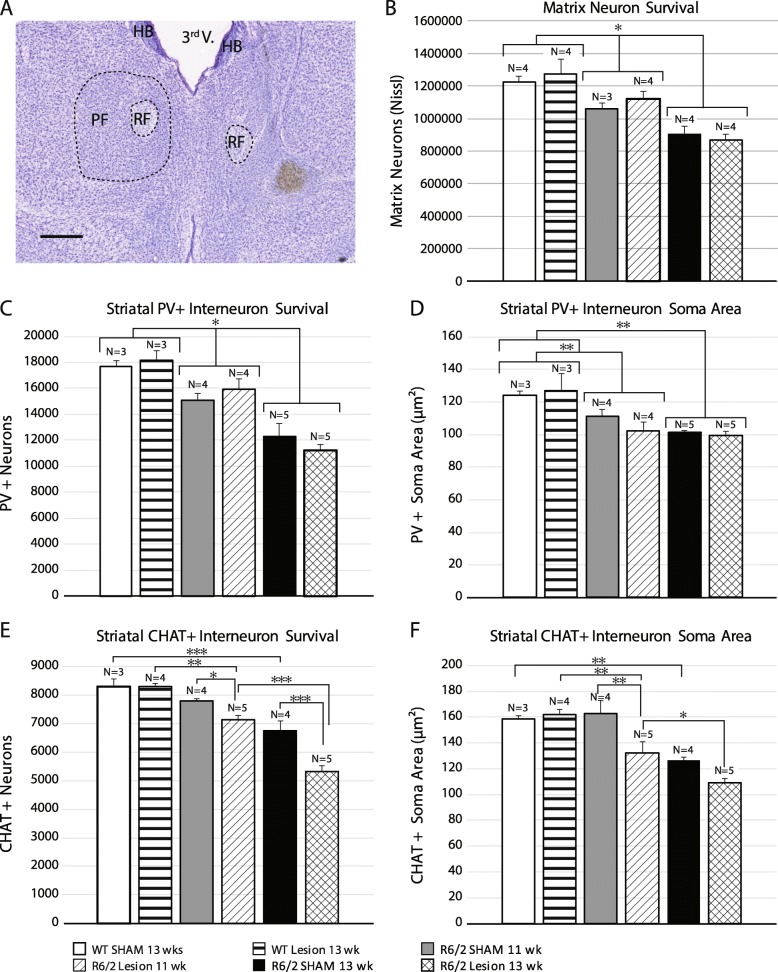
Analysis of number and size of striatal neuron subtypes after PF lesions. These subtypes are known to receive PF input. (**a**) A typical PF lesion in an R6/2 mouse (scale bar: 0.5 mm). (**b**) Unbiased stereology using the optical fractionator reveals loss of matrix neurons in R6/2 mice at 11 wks with further loss at 13 wks. PF lesions do not alter neuron number in the striatal matrix compartmentin either WT or R6/2 mice. (**c, d**) Unbiased stereology analysis of striatal PV+ interneurons using the optical fractionator (**c**) or the nucleator (**d**) reveals progressive cell loss and atrophy in R6/2 vs. WT mice, with no effect of PF lesions. (**e, f**) Optical fractionator cell counts (**e**) and nucleator-derived soma area (**f**) of ChAT + interneurons show earlier, more severe and progressive cell loss and atrophy in PF lesioned R6/2 mice compared to sham-lesioned R6/2 mice at both 11 wks and 13 wks. Morphology of ChAT + interneurons is not altered by PF lesions in WT mice. Scale bar: 250 μm. A 2-way between subject ANOVA was applied to each data set followed by a Tukey HSD post hoc test; * *p* < 0.05, ** *p* < 0.01, *** *p* < 0.001. Abbreviations: PF = Parafascicular, FR = Fasciculus Retroflexus, 3rd V. = 3rd Ventricle, HB = Habenula

The number of matrix neurons in R6/2 mice undergoes a significant and progressive reduction over time compared to WT mice, and there is no effect of PF lesions (Fig. 3b, w (23) = 0.967, *p* = 0.72, F(GenotypeXLesion)_2,17_ = 0.49, *p* = 0.62, F(Lesion)_1,17_ = 0.27, *p* = 0.61, F(Genotype)_2,17_ = 23.45, *p* = 0.00001. post hoc: WT vs 11 wks R6/2, *p* = 0.03, WT vs 13 wks R6/2, *p* = 0.0002, 11 wks R6/2 vs 13 wks R6/2, *p* = 0.003). As with neuronal counts, there is a significant reduction in the soma area in R6/2 mice at 11 and 13 wks compared to WT, with no effect of PF lesions (Additional file 1: Figure S1, W(23) = 0.981, *p* = 0.90, F(GenotypeXLesion)_2,17_ = 2.82, *p* = 0.09, F(Genotype)_2,17_ = 48.78, *p* < 0.00001, F(Lesion)_1,17_ = 1.13, *p* = 0.30).

The number of neurons in striosomes is significantly reduced in R6/2 mice compared to WT at 13 wks, but not at 11 wks. PF lesions did not alter striosome neuron number in any group (Additional file 1: Figure S2**,** W(23) = 0.986, *p* = 0.63 F(GenotypeXLesion)_2,17_ = 0.31, *p* = 0.74, F(Lesion)_1,17_ = 1.00, *p* = 0.33, F(Genotype)_2,17_ = 10.65, *p* = 0.001). Striosomal soma area was significantly decreased in 11 and 13 week old R6/2 mice compared to WT but there was no significant effect of PF lesions (Additional file 1: Figure S3, W(23) = 0.963, *p* = 0.5362, F(GenotypeXLesion)_2,17_ = 0.69, *p* = 0.51, F(Genotype)_2,17_ = 43.06, *p* < 0.00001, F(Lesion)_1,17_ = 4.97, *p* = 0.04). In summary, striatal neuronal loss occurs in R6/2 mice with onset in the matrix compartment, followed by loss in both compartments at late time-points. However, striatal projection neuron loss is not altered in either compartment by early PF lesions.

To determine if TS afferents sustain PV striatal interneurons in the face of degenerative stress [48], neurons were quantified by unbiased stereology in R6/2 mice at 11 and 13 wks following PF lesions at 4 wks. There is a significant and progressive reduction in the number of PV+ neurons in both PF lesioned and sham-lesioned R6/2 mice at 11 and 13 wks compared to WT. However, PF lesions did not alter PV+ interneuron number (Fig. 3c, w (24) = 0.965, *p* = 0.56, F(GenotypeXLesion)_2,18_ = 0.97, *p* = 0.40, F(Lesion)_1,18_ = 0.017, *p* = 0.90, F(Genotype)_2,18_ = 34.36, p < 0.00001, post hoc*:* WT vs 11 wks R6/2, *p* = 0.02; WT vs 13 wks R6/2, *p* = 0.0002; 11 wks R6/2 vs 13 wks R6/2, *p* = 0.0003). Furthermore, PV+ soma area was reduced with age in R6/2 mice, without an additional effect of PF lesions (Fig. 3d, w (24) = 0.978, *p* = 0.86, F(GenotypeXLesion)_2,18_ = 0.86, *p* = 0.44; F(Lesion)_1,18_ = 0.42, *p* = 0.53, F(Genotype)_2,18_ = 16.72, *p* = 0.00008, post hoc: WT vs 11 week *p* = 0.003; WT vs 13 week R6/2 p = 0.0002). Thus, PV+ cells undergo progressive atrophy and cell loss in R6/2 mice at late stages, but this degeneration is not affected by TS deafferentation.

The TS is the predominant source of glutamatergic input to striatal cholinergic interneurons [30, 49–52] and modulates their physiology [53]. To determine if loss of trophic support from the TS system alters striatal cholinergic neuron survival in R6/2 mice, we quantified choline acetyltransferase (ChAT) + cell number and soma size at 11 and 13 wks following PF lesions at 4 wks. Compared to WT mice, sham-lesioned R6/2 mice show a relative resistance to cholinergic neuron loss compared to MSNs or PV+ interneurons, with detectable reduction in numbers occurring at 13 wks, but not at 11 wks (Fig. 3e, w (25) = 0.982, *p* = 0.92, F(GenotypeXLesion)_2,19_ = 5.81, *p* = 0.01, post hoc: WT sham vs 13 wks R6/2 sham *p* = 0.0005; 11 wks R6/2 sham vs 13 wks R6/2 sham *p* = 0.005). PF lesioned R6/2 mice show accelerated loss of cholinergic neurons by 11 wks compared to both PF lesioned WT mice and sham-treated R6/2 mice, with further neuronal loss noted in PF lesioned R6/2 mice at 13 wks (Fig. 3e, post hoc: WT lesion vs 11 week R6/2 lesion, *p* = 0.002; WT lesion vs 13 wks R6/2 lesion, *p* = 0.0001; 11 wks R6/2 lesion vs 13 wks R6/2 lesion, *p* = 0.0002; 11 week R6/2 lesion vs 11 wks R6/2 sham *p* = 0.03, 13 wks R6/2 lesion vs 13 wks R6/2 sham, p = 0.0002). PF lesions did not induce cholinergic cell loss in WT mice. Thus, cholinergic degeneration occurs at a very late timepoint in sham R6/2 mice (13 wks) while PF lesioned R6/2 mice show an accelerated cholinergic cell loss at 11 wks that progresses at 13 wks.

Cholinergic soma area is also reduced at 13 wks in R6/2 compared to WT mice. PF lesions in R6/2 mice are associated with a further decrease in soma area beginning at 11 wks, which progresses by 13 wks (Fig. 3f, w (25) = 0.98, *p* = 0.90, F(GenotypeXLesion)_2,19_ = 3.36, *p* = 0.05, post hoc: WT sham vs 13 wks R6/2 sham *p* = 0.003; 11 wks R6/2 sham vs 13 wks R6/2 sham *p* = 0.02; WT lesion vs 11 wks R6/2 lesion, *p* = 0.009; WT lesion vs 13 wks R6/2 lesion *p* = 0.0002; 11 wks R6/2 lesion vs 13 wks R6/2 lesion *p* = 0.04). In summary, there is more severe cholinergic neuron atrophy in PF lesioned R6/2 mice compared to sham-lesioned R6/2 mice at 11 wks (p = 0.003), with further atrophy noted at 13 wks.

### Cholinergic interneuron loss following intrastriatal injection of immunotoxin

**Fig. 4:**
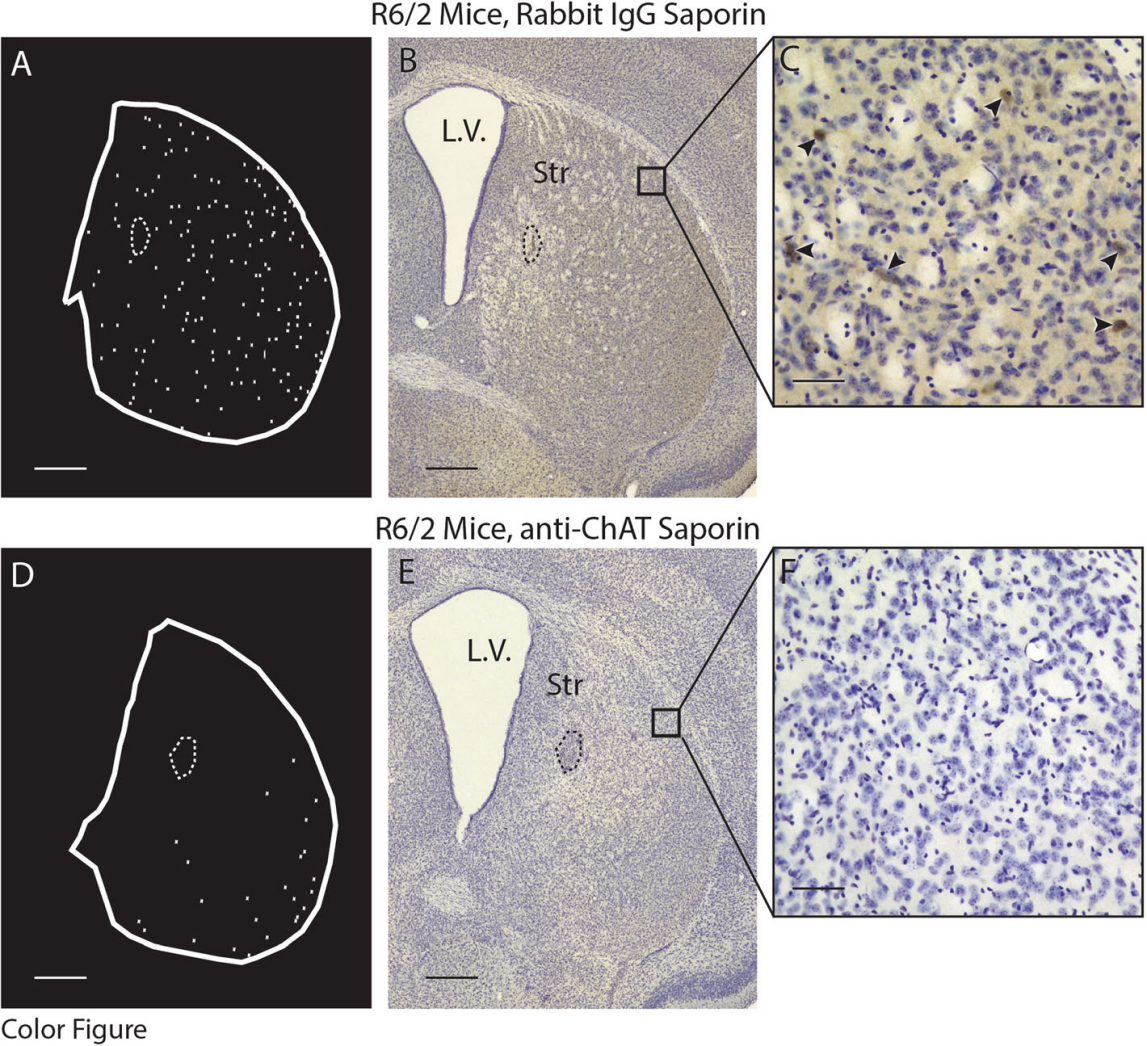
Cholinergic interneuron distribution in representative striatal sections of R6/2 mce 7 wks after intrastriatal injection of either anti-ChAT-saporin immunotoxin or Rabbit IgG-saporin (control). (**a, d**) Contours demonstrating the location of ChAT+ profiles in coronal sections of neostriatum. (**b, c, e, f**) Photomicrographs of corresponding sections immunohistochemically stained for ChAT and Nissl after treatment with either Rabbit IgG-saporin (**b**, 4X; **c**, 20X) or anti-ChAT-saporin (**e**, 4X; **f**, 20X). Arrowheads demonstrate ChAT+ striatal neurons. Stippled contours indicate the hemosiderin artifact from the injection site. Squares in (**b**) and (**e**) represent the area magnified in (**c**) and (**f**) respectively. Scale bars: A, B, D, E = 500 μm, C, F = 100 μm. Abbreviations: L.V. = Lateral Ventricle, Str = Striatum

**Fig. 5:**
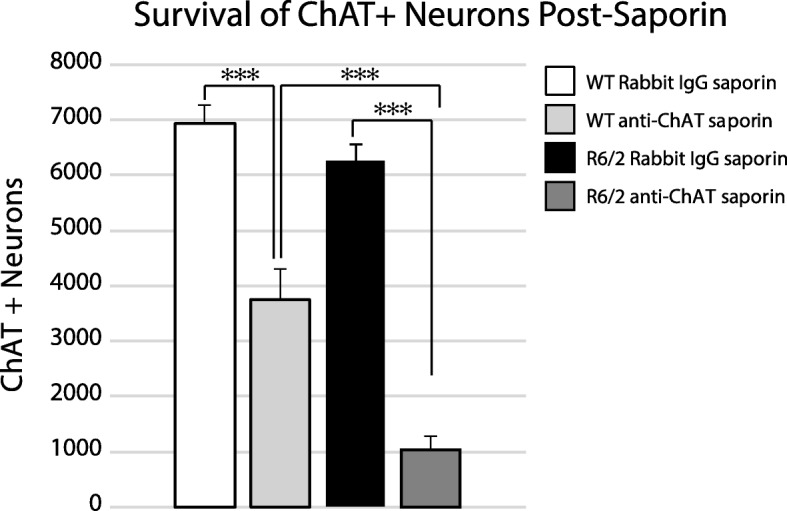
Unbiased stereological assessment of striatal cholinergic interneuron number 7 wks after intrastriatal injection of anti-ChAT-saporin immunotoxin or Rabbit IgG-saporin (control). ChAT+ neuron numbers were significantly reduced in both R6/2 (*p* = 0.0002) and WT mice (*p* = 0.0003) receiving cholinergic specific toxin compared to control saporin. The reduction in ChAT+ neurons was more pronounced in R6/2 compared to WT mice following exposure to anti-ChAT-saporin (*p* = 0.0004). A two-way between subject ANOVA was applied to the data, followed by a Tukey HSD post hoc test; *** *p* < 0.001. WT Rabbit IgG-saporin: *n* = 3; WT anti-ChAT-saporin: *n* = 3; R6/2 Rabbit IgG-saporin: *n* = 3; R6/2 anti-ChAT-saporin: *n* = 5

To determine if cholinergic neuron loss is associated with changes in motor phenotype, anti-ChAT conjugated saporin toxins were used to selectively ablate striatal cholinergic interneurons. Mice received intrastriatal injections of either anti-ChAT-saporin or Rabbit IgG-saporin (control saporin) at 4 wks of age and were euthanized at 11 wks (Fig. 4). There was a large reduction in the number of cholinergic neurons assessed using unbiased stereology in both R6/2 and WT mice injected with anti-ChAT -saporin (Fig. 5, W(14) = 0.895 *p* = 0.09, F(GenotypeXSaporin)_1,10_ = 8.08, p = 0.02; post hoc*:* anti-ChAT-saporin WT vs Rabbit-IgG -saporin WT: *p* = 0.0003, anti-ChAT-saporin R6/2 vs Rabbit IgG-saporin R6/2: p = 0.0002). The reduction in cholinergic number in anti-ChAT -saporin injected R6/2 mice was greater than in anti-ChAT-saporin injected WT mice (*p* = 0.004). Soma size of the surviving cells was not different among the four groups (Additional file 1: Figure S4, W(14) = 0.944 *p* = 0.4754, F(GenotypeXSaporin)_1,10_ = 0.46, *p* = 0.51, F(Genotype)_1,10_ = 3.28, *p* = 0.10, F(Saporin)_1,10_ = 0.23, *p* = 0.64). Thus, an intrastriatal anti-ChAT-saporin injection was effective in eliminating a substantial proportion of striatal cholinergic neurons in both WT and R6/2 mice. Moreover, striatal cholinergic neurons were significantly more vulnerable to the cholinergic immunotoxin in R6/2 compared to WT mice.

### The effect of striatal cholinergic ablation on motor behavior in R6/2 and WT mice

**Fig. 6:**
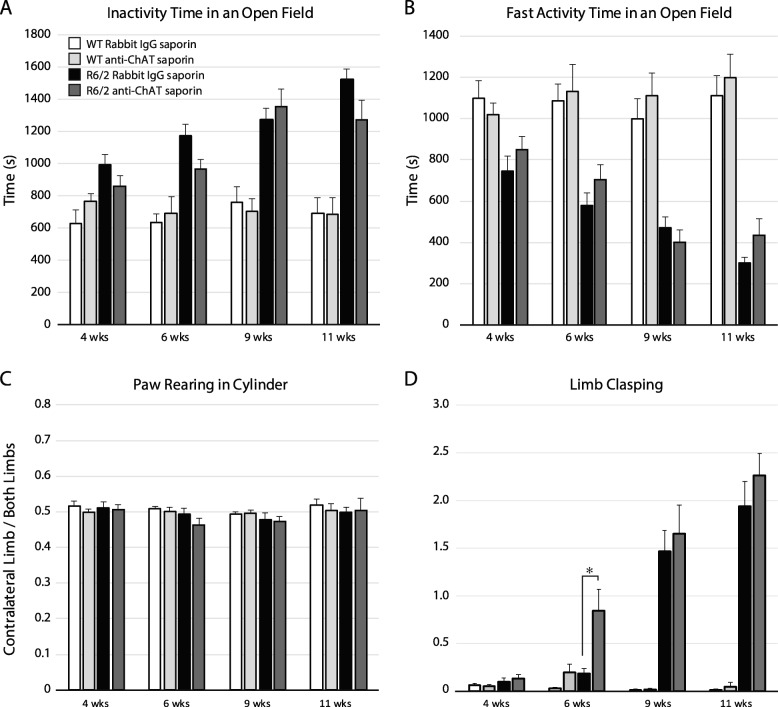
Characterization of motor behavior following unilateral striatal cholinergic ablation in R6/2 and WT mice. (**a**) Time spent at rest and (**b**) on rapid movements during a one-hour open field session demonstrating a decrease in spontaneous voluntary locomotor activity over time in R6/2 mice with no significant effect of anti-ChAT- or Rabbit-IgG-saporin injections. (**c**) Cylinder test assessing limb use asymmetry shows no effect of saporin injection on paw preference. (**d**) Evaluation of dystonia shows an increase in limb clasping at 6 wks in anti-ChAT saporin treated R6/2 mice compared to control-saporin treated R6/2 mice (*p* = 0.04). A 3-way non-parametric ANOVA was applied to each data set, followed by a post hoc Bonferroni correction; *p < 0.05. For all panels of Fig. 6, WT Rabbit-IgG-saporin: *n* = 9, WT anti-ChAT-saporin: *n* = 10, R6/2 Rabbit-IgG-saporin: *n* = 12, R6/2 anti-ChAT-saporin: *n* = 11

To determine the effect of striatal cholinergic ablation on spontaneous locomotor activity, anti-ChAT-saporin or Rabbit-IgG-saporin injected R6/2 and WT mice were placed in an open field for one hour at 4, 6, 9 and 11 wks. Compared to WT mice, R6/2 mice showed a decrease in spontaneous locomotion as revealed by increased time spent resting in an open field at 6,9 and 11 wks, with no effect of anti-ChAT-saporin injection (Fig. 6a, f (GenotypeXSaporinXTime) _3120_ = 2.50, *p* = 0.06, F(GenotypeXTime) _3120_ = 13.8, *p* < 0.0001, post hoc: R6/2 vs WT after 6 wks, all *p* < 0.005). Decreased time spent on fast activity reflected the rest time results (Fig. 6b F(TimeXGenotypeXSaporin) _3120_ = 2.77, *p* = 0.04; post hoc WT vs R6/2 after 6 weeks all p < 0.005, post hoc: all comparisons within genotype for Rabbit-IgG-saporin vs anti-ChAT-saporin were not significant). Thus, striatal cholinergic ablation does not affect spontaneous voluntary locomotor behaviour of R6/2 and WT mice.

The cylinder test assessing limb use asymmetry while performing vertical exploration revealed no differences in paw reaching for either of the saporin treated groups (Fig. 6c, w (60) = 0.974 *p* = 0.2354, F(GenotypeXSaporinXTime)_3142_ = 0.33, *p* = 0.80, F(GenotypeXSaporin)_1142_ = 0.001, p = 0.80, F(TimeXSaporin)_3142_ = 0.14, *p* = 0.94, F(TimeXGenotype)_1142_ = 0.51, *p* = 0.67). Thus, unilateral striatal cholinergic ablation does not induce a paw preference during voluntary movement in either R6/2 or WT mice.

To determine if cholinergic ablation affected the development of the dystonic phenotype in R6/2 mice, limb clasping was assessed at 4, 6, 9 and 11 weeks. Dystonic clasping behaviour with aging worsened in R6/2 mice in both anti-ChAT-saporin and Rabbit IgG-saporin treated groups. There was a significant increase in clasping at 6 wks in anti-ChAT-saporin treated R6/2 mice compared to control anti-Rabbit IgG-saporin treated R6/2 mice (Fig. 6d, f (TimeXGenotypeXSaporin)_3114_ = 4.31, *p* = 0.006; post hoc: 6-week anti-chat saporin R6/2 vs 6-week control anti-Rabbit IgG-saporin R6/2 *p* = 0.04). Both anti-ChAT-saporin and Rabbit-IgG-saporin injected WT mice exhibited virtually no clasping behaviour. Thus, striatal cholinergic ablation significantly accelerates the development of a dystonic phenotype in R6/2 mice.

## Discussion

Dysfunctional striatal afferents may play an important role in mechanisms leading to motor symptoms in HD [24, 31, 32, 54]. Here we demonstrate that the major source of thalamostriatal (TS) projections, the CM-PF complex in primates or the PF in rodents, degenerates in the R6/2 mouse model of HD. Furthermore, depriving the R6/2 striatum of TS inputs prior to onset of motor signs results in an acceleration of dystonic involuntary movements. Complex voluntary motor behaviours such as spontaneous paw reaching are also impaired following TS deafferentation in R6/2 compared to WT mice. The time course of worsening of spontaneous locomotion in an open field is not altered after unilateral TS lesions. Morphological analysis of degenerating striatal neurons indicates that the cholinergic interneuron subtype is especially vulnerable to TS denervation in the R6/2 mouse. In contrast, the time course of loss of MSNs and parvalbumin-positive interneurons is unaltered following PF lesions in R6/2 mice. Finally, induction of striatal cholinergic loss in the R6/2 striatum using immunotoxins reproduces the acceleration of dystonia seen after TS denervation in R6/2 mice, suggesting that abnormal TS-cholinergic interactions are an important contributor to the dystonia phenotype in HD.

### The role of afferents in loss of striatal neurons in HD

In HD, *mhtt* protein is expressed throughout the organism, but the striatum is especially vulnerable to degeneration [2]. Striatal neurons are likely lost due to multiple cell autonomous mechanisms [8, 10–13, 15]. Striatal afferents may contribute to cell non-autonomous mechanisms of neuron dysfunction or death by loss of anterograde neurotrophic support [16, 55, 56], excitotoxicity related to abnormal ionotropic receptor signaling [12, 57–59] or abnormal synaptic transmission [31, 60].

Glutamatergic afferents from the cerebral cortex to the striatum may participate in neuronal loss in HD by inducing excitotoxicity [12, 57–59]. Depriving the HD striatum of cortical afferents in the R6/2 model using lesions restricted mainly to the motor cortex appears to protect neurons sampled from the dorsolateral striatum from atrophy, although neuronal counts were not available [14]. These lesioned mice also showed reduced clasping [14], a finding that may be confounded by pyramidal effects from lesioning the motor cortex. An excitotoxic role for corticostriatal (CS) glutamatergic afferents on striatal MSNs in HD was suggested. This is in keeping with previous work by several groups indicating that aberrant calcium signaling through extra-synaptic NMDA receptor (NMDAR) stimulation and increased sensitivity of NMDARs is linked to MSN excitotoxicity in HD [9–12, 15, 57–59]. On the other hand, CS afferents are potentially protective for MSNs, an effect that may be mediated by synaptic glutamatergic mechanisms [61, 62] or by anterograde neurotrophin-related effects [34, 36, 55, 56, 63].

In comparison to the cerebral cortex, relatively little is known of the role of the other major source of glutamatergic striatal afferents, the posterior intralaminar nuclei, in mechanisms of striatal dysfunction in HD. In vivo imaging of patients demonstrates that thalamic atrophy occurs early in the course of HD [23], and autopsy studies provide evidence for significant loss of CM-PF neurons [24]*.* Ultrastructural evidence from the Q140 heterozygous mouse model of HD provides morphological evidence for early TS dysfunction, since TS synapses on MSNs are lost by one month, whereas loss of corticostriatal synapses is observed relatively late, at one year [32]. Recent work in 9–12 week old R6/2 mice also suggests abnormal morphology of TS inputs [64]. The present work demonstrates that PF neurons, the main source of TS inputs, are lost in R6/2 mice coincident with the onset of detectable neurodegenerative changes in the neostriatum on Nissl stains [34]. Indeed. PF neuron atrophy is already detected 9 wks, prior to significant striatal neuronal loss. There is progressive loss of PF neurons at 11 and 13 wks correlating with worsening dystonia and other locomotor deficits. Interestingly, the early significant reduction of average neuronal soma size at 9 wks is followed by apparent normalization of average soma size at 11 wks. Neuronal loss and average cell size do not necessarily correlate. Indeed, as degeneration progresses, it is expected that neurons with decreased cell size will be lost preferentially. As a result, there would be a relative abundance of larger neurons with apparent normalization of cell size. With further progression of degeneration, the remaining neurons that were initially spared may also degenerate resulting in the observed reduction in soma area at 13wks. Alternatively, the degenerating PF neurons may represent a specific subpopulation. For example, different cellular subpopulations within the mouse PF may provide preferential inputs to MSNs or striatal cholinergic interneurons [65]. It would be of interest to determine whether specific intralaminar thalamic subpopulations degenerate in post-mortem HD brains and in HD models.

Another important differentiating factor between glutamatergic striatal afferents is revealed by physiological studies in slice preparations indicating that the PF preferentially elicits NMDA currents in MSNs while CS afferents evoke a higher proportion of AMPA- mediated post-synaptic currents [26, 66]. The apparently larger contribution of NMDA mediated post-synaptic currents from PF inputs compared to CS afferents [26, 66], may suggest a differential role for the TS or CS in excitotoxicity [64, 67]. Differential inputs to patch and matrix compartments that comprise the striatal mosaic may provide a clue to differences in thalamic or cortical-derived afferent effects on MSN survival in HD. Unlike the cerebral cortex which innervates all MSNs, the PF provides dense afferents almost exclusively to the matrix compartment of the striatum [25–27]. Therefore, potential excitotoxicity from the PF would be expected to have differential effects on MSNs in either compartment. Alternatively, TS afferents may also provide a sustaining role for vulnerable striatal neurons in HD [34], analogous to their trophic survival role in normal striatal development [36]. Indeed, BDNF is enriched in PF neurons [36, 68], and there is an early reduction in BDNF mRNA in striatal afferents including in the PF of R6/2 mice [34]. Furthermore, the ability to activate striatal TrkB receptors in the R6/2 striatum is impaired [13]**.** Importantly, the present findings indicate that early TS lesions in R6/2 mice have no significant effect on MSN size or number using unbiased stereology performed separately on either patch or matrix compartments of sham and lesioned R6/2 mice. These findings suggest that loss of projection neurons in HD likely involves a complex interplay between neurotrophic, excitotoxic and cell autonomous mechanisms, and loss of glutamatergic TS afferents is not a major factor determining survival of MSNs in the HD striatum.

### Vulnerability of specific interneuron subtypes

Although striatal interneuron subtypes comprise only 5–10% of the striatal population, they are important modulators of striatal function in health and disease states [5, 37, 53, 69–76]. Striatal interneurons include cholinergic neurons, and GABAergic subtypes that express somatostatin, parvalbumin, or calretinin [76]. Striatal interneurons modulate MSNs via local synapses, and also at a distance across patch/matrix boundaries [77, 78]*.* In rodents, the PF contributes only a small proportion of excitatory synapses to striatal PV neurons [29, 74]. In contrast, striatal PV interneurons receive dense asymmetric inputs from the cerebral cortex [74, 79] suggesting they may be more sensitive to pathological changes affecting the cortex rather then the PF in HD. Early work suggested that striatal PV interneurons may be spared in HD, [80] but more recent findings in autopsied HD brains indicate an important reduction in PV interneurons [5]. The present results provide stereological evidence for a decrease in soma size and number of striatal PV interneurons in the R6/2 model of HD. Early PF lesions in the R6/2 model do not accelerate the time course of degeneration of PV interneurons. As with MSNs, degeneration of PV neurons is likely due to a combination of cell autonomous and non-autonomous mechanisms [48, 81], but the TS projection does not play a major survival role for PV interneurons in the face of neurodegenerative stress in HD.

Cholinergic interneurons make up 1% of all striatal neurons, synapse on most MSNs and other interneurons, and modulate dopaminergicand glutamatergic terminals in the striatum [72]. Ultrastructural studies indicate that the predominant glutamatergic input to cholinergic interneurons is from the posterior intralaminar nuclei in rodents and monkeys [30, 49–52]. Although physiological and viral-based tracing studies [82, 83] suggest that cholinergic interneurons may receive cortical input, there is little ultrastructural evidence for inputs from the cerebral cortex in rodents [28]. Classically, cholinergic interneurons were thought to be spared in HD [4]. However, recent evidence points to significant striatal cholinergic dysfunction in HD patients, including reduced synthetic and vesicular proteins [84, 85], and decreased ChAT+ cell numbers [6]. Several electrophysiological studies have shown abnormal cholinergic responses to afferent stimulation and decreased acetylcholine release in slice preparations in R6/2 or Q175 mouse models [54, 86–88]. In the R6/1 mouse model of HD, striatal vesicular acetylcholine transporter, and ChAT mRNA and protein concentrations are reduced in tissue lysates, and *mhtt* aggregates accumulate in cholinergic neurons [84]. Ultrastructural evidence in the Q140 mouse model of HD indicates that striatal cholinergic interneurons have a decreased number of TS synapses, reduced cell diameter and fewer dendritic branches [31]. In keeping with this work, ex vivo brain slices derived from the Q175 mouse model of HD show decreased synaptic facilitation at cholinergic targets in response to PF stimulation [54]. The present results from R6/2 mice suggest that neuronal degeneration in the PF occurs early in the course of HD, and therefore contributes to loss of TS synaptic integrity and function [64]. The observed loss of PF neurons precedes cholinergic neuron atrophy and cell loss which normally only occurs at late timepoints suggesting a relative resistance of cholinergic neurons to degeneration in HD. Early PF lesions accelerate the atrophy and loss of cholinergic neurons in R6/2 mice, suggesting that these neurons are especially dependent on sustaining thalamic input in the face of *mhtt* related neurodegenerative stress.

Multiple mechanisms may underlie the differential vulnerability of striatal cholinergic interneurons to TS deafferentation compared to other striatal populations. The fact that the glutamatergic TS system provides more prominent input to cholinergic interneurons [30, 49, 50, 52] compared to PV interneurons [29, 74] may explain their sensitivity to TS deafferentation in R6/2 mice through both glutamatergic and trophic factor receptor dependent mechanisms. For example, cholinergic interneurons express lower levels of ionotropic NMDA-2A and metabotropic GluR1/5 glutamate receptors [89] then other striatal cell types, but maintain high NMDA-2B expression [90–92]. Signaling from mGLUR5 and synaptic NMDA receptors enriched in NMDA-2A subunits can stabilize mitochondrial membranes and promote cell survival, [61, 62] while neurotoxic extrasynaptic NMDA receptors rich in NMDA-2B subunits contribute to mitochondrial failure and cell death in MSNs in various HD models [9, 11, 15, 57, 59–61]. In keeping with this evidence, the present in vivo results demonstrate striatal cholinergic interneurons in R6/2 mice are more susceptible to a mitochondrial toxin than WT neurons suggesting that they are more vulnerable to cellular energy failure.

In addition to glutamatergic modulation of cell death, neurotrophins may also play an important role in cholinergic neuron vulnerability to TS loss in HD. The neurotrophin brain-derived neurotrophic factor (BDNF) promotes forebrain cholinergic neuron maintenance, growth [93], and survival [94, 95]. In particular, ChAT+ striatal neurons express both TrkA and TrkB receptors [96, 97] and contain BDNF protein [98]. Given that the striatum lacks BDNF mRNA [68, 99–103], the BDNF protein in ChAT+ cells may derive from post-synaptic internalization and endosomal trafficking of BDNF released from afferents [104–110]. Endosomal trafficking of TrkB/BDNF complexes towards the soma provides trophic support to neurons, is regulated by *htt* and is reduced in the presence of *mhtt* [108, 111–114]. Since a higher proportion of cholinergic neurons express *htt* compared to other striatal subpopulations [98, 115], they may be especially vulnerable to loss of BDNF. Importantly, the TS system is the main source of glutamatergic afferents to cholinergic neurons [30, 49–52] is enriched in BDNF mRNA [34, 68], and PF lesions reduce striatal BDNF-TrkB signaling in neonatal rodents [36]. The PF may therefore contribute to the relative resistance to degeneration of cholinergic neurons. Loss of BDNF following PF lesions or degeneration in HD may make cholinergic neurons more vulnerable to degeneration in HD.

### Implications of thalamostriatal and cholinergic dysfunction for dystonia and HD

Dystonia can be a motor feature of both hypokinetic and hyperkinetic disorders including primary and secondary dystonic syndromes, and other neurodegenerative diseases [1116–118]. Secondary dystonia can occur following lesions in different parts of the thalamic, cerebellar or basal ganglia network [119–124]. Dystonia is also a common symptom in HD, and worsens with disease progression, but appears not to correlate with chorea or bradykinesia [1]. Clasping behaviour is considered a surrogate for dystonia in rodent models as it mimics the sustained muscle contractions and abnormal postures seen in humans [116]. Clasping behaviour occurs in many animal models of HD and primary dystonia [34, 47, 71, 125]. Furthermore, clasping behavior is well studied in the R6/2 mouse and worsens significantly as the model progresses [34], similar to the age related increase in dystonia seen in HD patients [1].

Basal ganglia, cerebellar, brainstem and cortical dysfunction are proposed in both human dystonia and in the many animal models exhibiting clasping behaviours reminiscent of dystonia [125, 126]. Interrogation of different components of the striatal micro-circuitry in animal models allows better understanding of dystonia. DYT1 mouse models of primary dystonia show decreased intrastriatal dopamine release possibly due to reduced nicotinic cholinergic tone [127], and paradoxical dopamine D2 receptor mediated excitation of cholinergic neurons [128, 129]. These altered cholinergic-dopaminergic interactions in DYT1 mice impair long-term depression in MSNs and increase corticostriatal synaptic long-term potentiation, leading to abnormal striatal output [126, 127, 129, 130]. These synaptic deficits may even occur early in brain development, as mice with selective forebrain DYT1 knockout show an early clasping phenotype associated with post-natal loss of cholinergic striatal interneurons and decreased striatal acetylcholine release [47]. Similar changes in striatal cholinergic micro-circuitry are described in HD models. These include: the inability of striatal cholinergic cells to undergo long-term potentiation with an associated inability of MSNs to undergo long-term synaptic depression [87], decreased acetylcholine release [86, 88], and increased MSN and cholinergic responses to cortical excitation [54, 64]. These abnormalities are compounded in HD by atrophy and loss of striatal cells [2, 5–7].

The posterior intralaminar nuclei are important drivers of cholinergic activity in the normal striatum [37, 53, 69, 70, 73, 75, 131, 132]. Cholinergic neurons modulate long-term plasticity of MSNs by regulating dopamine and glutamate co-release onto MSNs through pre-synaptic acetylcholine receptors on glutamatergic and dopaminergic terminals [132]. More specifically, the pause-response of cholinergic neurons to TS stimulation, which is mediated by D2-receptors on cholinergic cells as well as presynaptic nicotinic receptors on dopaminergic terminals, helps to transiently inhibit both direct and indirect pathway MSNs responses to cortical stimulation and then later facilitate post-synaptic cortical glutamatergic excitation of indirect pathway neurons, thereby preferentially driving the network towards action cessation [53]. Loss of the PF-cholinergic mediated tuning of striatal projection neurons leads to an imbalance between competing basal ganglia pathways and is thought to impair saliency estimation and motor program selection [69, 70, 73, 131, 133], and contribute to the generation of dystonia [126]. Indeed, TS-cholinergic deficits have been shown in HD and dystonia models. For example, in a DYT1 mutant mouse, the normal pause-response is replaced by erratic firing of cholinergic cells to TS stimulation [130] and in the Q175 HD mouse, there is reduced TS synaptic facilitation of cholinergic interneurons and loss of the normal pause-spike response to TS stimulation [54]. The present work demonstrates that lesioning either the TS system or striatal cholinergic interneurons exacerbates dystonia in the R6/2 HD mouse. Altogether these findings suggest that dysfunction of both the TS system and loss of cholinergic interneurons plays an important role in the generation of dystonia in HD and in primary dystonia models.

In addition to the striatum and the TS system, the cerebellum is proposed as an important part of the dystonia network. Evidence from imaging studies suggests reduced cerebellar activity, degeneration of the cerebello-thalamocortical pathway and abnormal cerebellar sensorimotor integration in dystonia patients [134–136]. HD patients show cerebellar degeneration that correlates with a worse motor score [2, 137, 138]. Furthermore, models which have a severe clasping phenotype such as R6/2 and Hdh100 HD mice also demonstrate a loss of Purkinje cells at late timepoints [139, 140].

The intralaminar nuclei receive afferents from deep cerebellar nuclei and form a disynaptic link between the basal ganglia and the cerebellar nuclei [141–143]. The output of the cerebello-thalamic circuit plays an important role in saliency estimation and action selection [69, 70, 73, 131]. Similar to the effect of unilateral cerebellar lesions in rats [144], PF lesions in the present work led to decreased spontaneous contralateral paw use in both WT and R6/2 mice when exploring a cylinder. This provides evidence for a role for the TS system in evaluation of salient sensory information and appropriate motor program selection. Furthermore, both thalamic and cerebellar strokes lead to secondary dystonia in susceptible individuals [119–124]. Atrophy and cell loss in the TS system and the cerebellum [2, 24, 137] may therefore contribute to the dystonia network in HD. TS afferents degenerate in R6/2 mice expressing a dystonic clasping phenotype, and early PF lesions in R6/2 mice lead to a worsening of dystonia. We therefore propose that TS degeneration, with downstream pathology at cholinergic targets, plays an important part in the network leading to expression of dystonia in HD and possibly in other dystonic syndromes.

## Conclusion

Thalamostriatal afferents provide important trophic support to striatal cholinergic neurons in Huntington’s disease. Furthermore, pathological dysfunction of the TS system and cholinergic interneurons is closely linked to the generation of a dystonic phenotype in HD models. This work provides a new understanding of mechanisms of striatal degeneration and motor symptoms in HD, and may pave the way for development of effective therapies for those affected by this currently incurable neurodegenerative disorder.

## Additional file

**Additional file 1: ** Experimental Timeline. **Figure S1.** Matrix Neuron Soma Area in WT and R6/2 mice. **Figure S2.** Striosome cell count in WT and R6/2 mice. **Figure S3**. Striosome Neuron Soma Area in WT and R6/2. **Figure S4.** CHAT+ Cell Soma Area in Saporin Treated Animals

## Data Availability

The data analysed during the current study are available from the corresponding author on reasonable request.

## Abbreviations

3rd V.: 3rd ventricle
AMPA: α-amino-3-hydroxy-5-methyl-4-isoxazolepropionic acid
BDNF: Brain-derived neurotrophic factor
CC: Corpus callosum
ChAT: Choline acetyltransferase
CM-PF: Centromedian-parafascicular
FR: Fasciculus retroflexus
HB: Habenula
HD: Huntington’s disease
L.V.: Lateral ventricle
*mhtt*: Mutant *huntingtin* gene
MSNs: Medium spiny projection neurons
NMDA: N-methyl D-aspartate
NMDAR: NMDA receptor
PF: Parafascicular
PV: Parvalbumin
Str: Striatum
Trk: Tyrosine kinase receptor
TS: Thalamostriatal
wks: Weeks
WT: Wildtype
