# Supplementary material for: Thalamostriatal degeneration contributes to dystonia and cholinergic interneuron dysfunction in a mouse model of Huntington’s disease
Source: Acta Neuropathol Commun. 2020 Feb 7;8:14. doi: 10.1186/s40478-020-0878-0 (PMC7007676; doi:10.1186/s40478-020-0878-0)
Supplement: Supplementary file 1 — Additional file 1: Experimental Timeline. Figure S1. Matrix Neuron Soma Area in WT and R6/2 mice. Figure S2. Striosome cell count in WT and R6/2 mice. Figure S3. Striosome Neuron Soma Area in WT and R6/2. Figure S4. CHAT+ Cell Soma Area in Saporin Treated Animals [file 40478_2020_878_MOESM1_ESM.pdf]

Supplementary Materials

Title: Thalamostriatal Degeneration Contributes to Cholinergic Interneuron Loss and Dystonia in a Huntington's Mouse Model

Journal Title : Acta Neuropathologica Communications

Authors: Gabriel Crevier-Sorbo, Vladimir V. Rymar, Raphael Crevier-Sorbo, Abbas F. Sadikot

Author Affiliation: Department of Neurology and Neurosurgery, Montreal Neurological Institute, McGill University, 3801 University street, H3A 2B4, Montreal, Quebec, Canada

Corresponding Author: Abbas F. Sadikot: abbas.sadikot@mcgill.ca, phone: 514 398 8542

Experimental Timeline

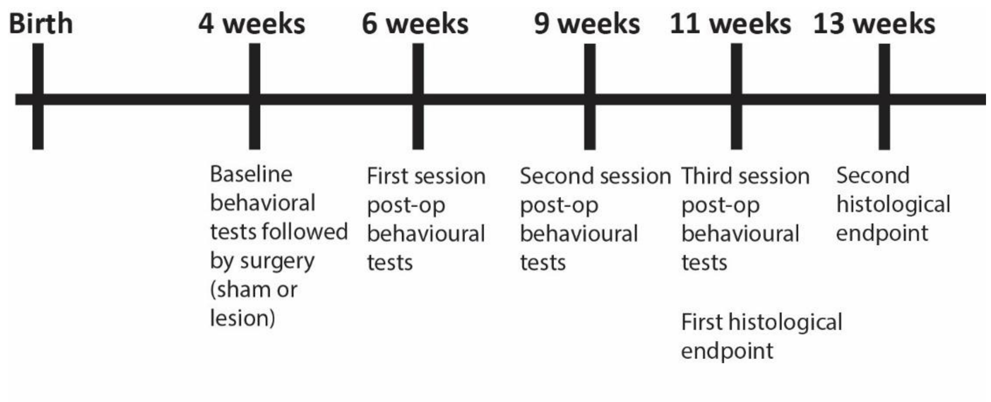

Supplementary Figures

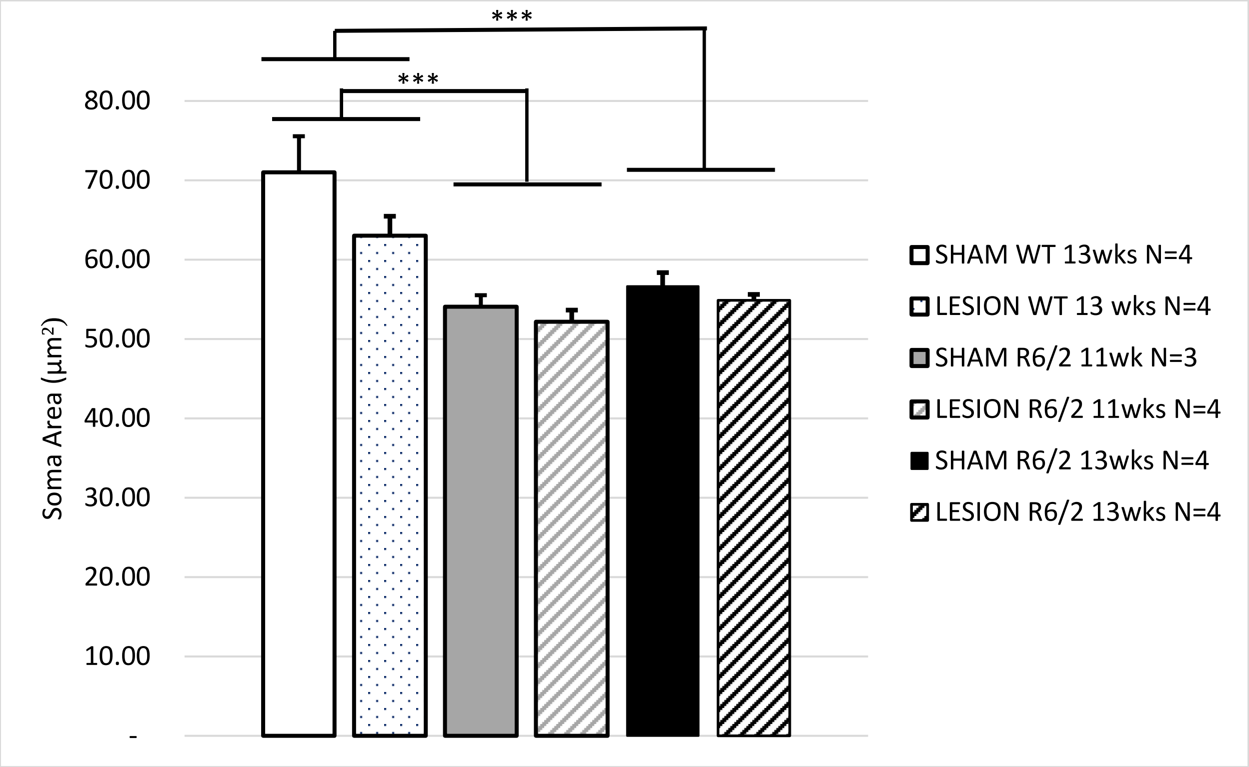

Supplementary Fig. 1: Matrix Neuron Soma Area in WT and R6/2 mice. Demonstrates a reduction in the R6/2 soma area of matrix neurons compared to WT at both 11 and 13wks with no significant effect of PF lesion. A 2-way between subject ANOVA was applied to the data and was followed by a Tukey HSD *post hoc* test where appropriate. ***$p<0.001$.

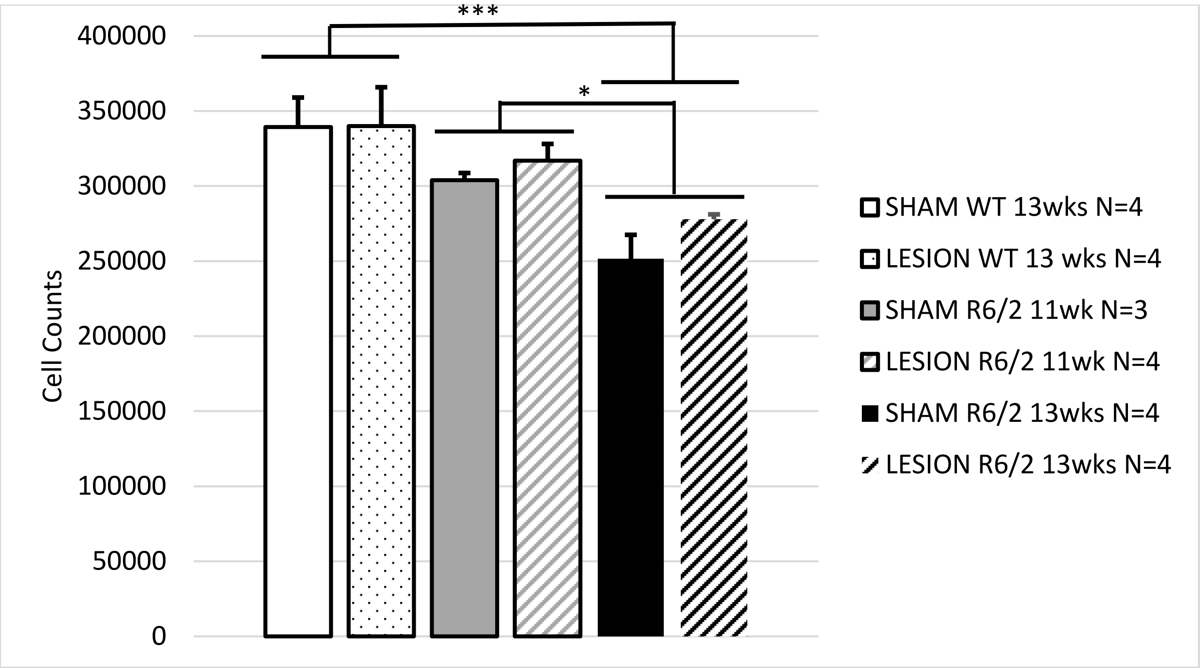

Supplementary Fig. 2: Striosome cell count in WT and R6/2 mice. Demonstrates a trend to reduction in number of striosome neurons is seen in R6/2 compared WT mice that was not significant (~8% decrease, p=0.23) at 11wks but was significant (p<0.05) at 13wks. There was no significant effect of thalamic lesion on striosome neuron number. A 2-way between subject ANOVA was applied, followed by a Tukey HSD *post hoc* test.*p<0.05; ***p<0.001.

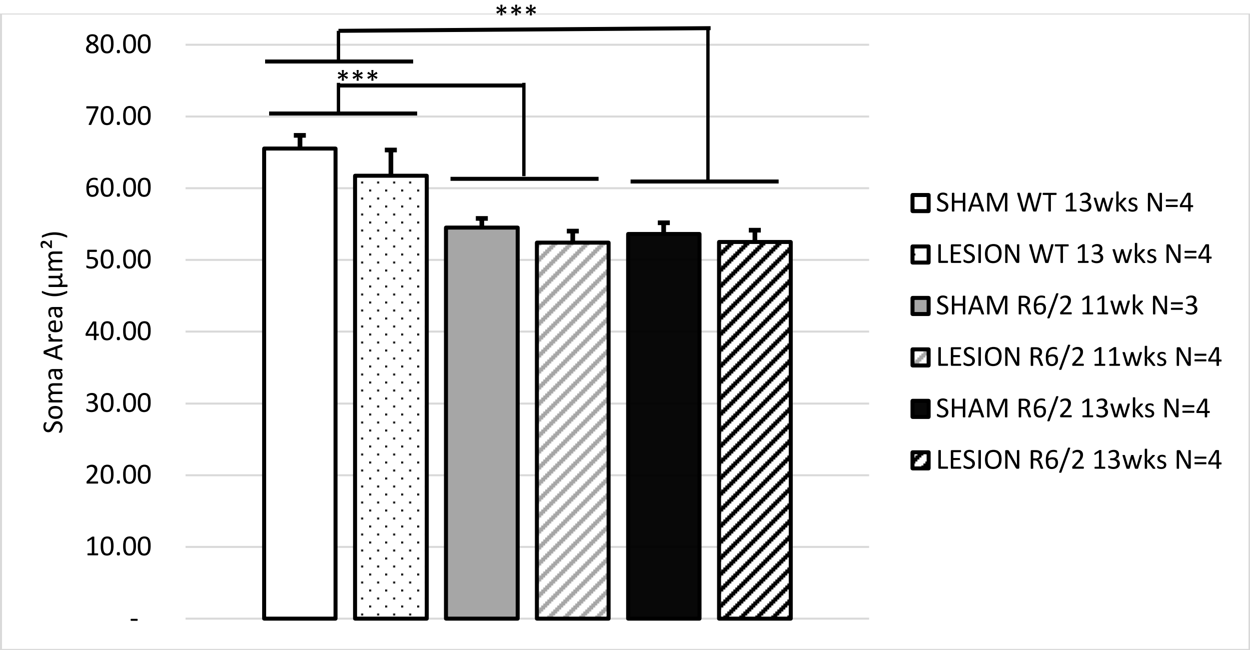

Supplementary Fig. 3: Striosome Neuron Soma Area in WT and R6/2. Demonstrates a reduction in soma area of striosome neurons in R6/2 animals at both 11 and 13 wks compared to WT. There is no significant effect of lesion on striosome neuron soma area. A 2-way between subject ANOVA was applied followed by a Tukey HSD *post hoc* test. ***p<0.001.

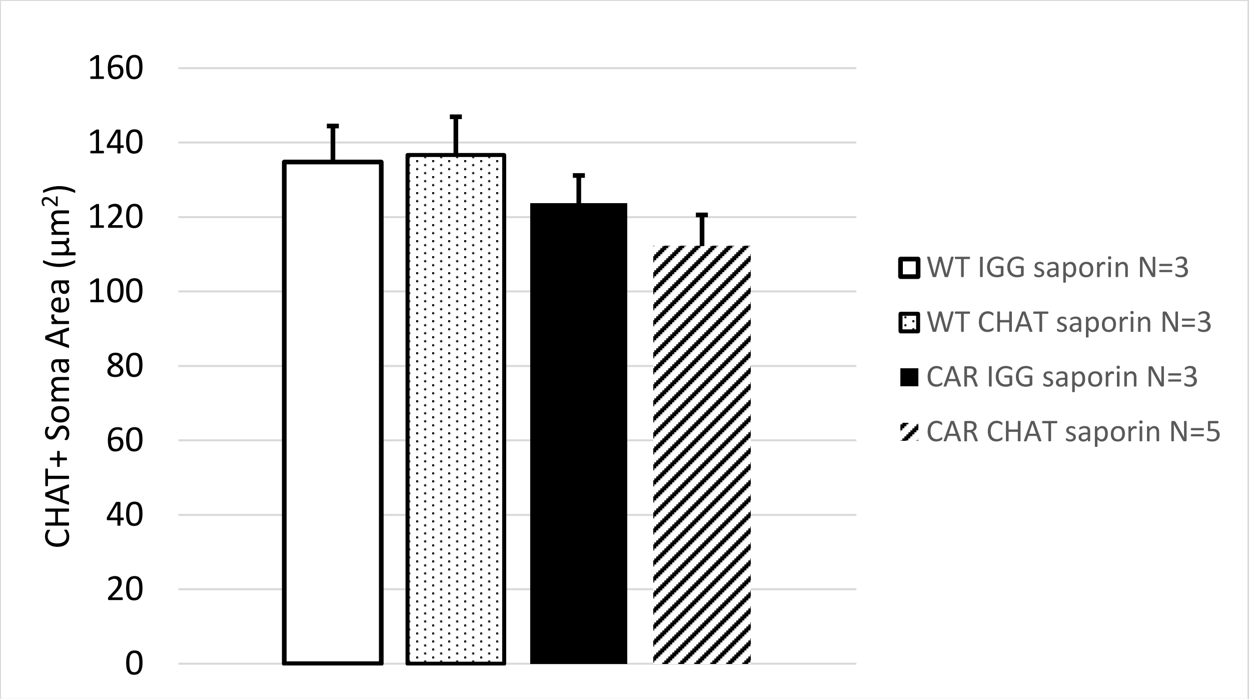

Supplementary Fig. 4: CHAT+ Cell Soma Area in Saporin Treated Animals. No significant difference between remaining CHAT+ cells in either WT or R6/2 animals treated with saporin toxins. A 2-way between subject ANOVA was applied to the data and was followed by a Tukey HSD *post hoc* test.
